# Early interventions for diabetes related tuberculosis associate with hastened sputum microbiological clearance in Virginia, USA

**DOI:** 10.1186/s12879-017-2226-y

**Published:** 2017-02-06

**Authors:** Yosra Alkabab, Suzanne Keller, Denise Dodge, Eric Houpt, Deborah Staley, Scott Heysell

**Affiliations:** 1 Division of Infectious Diseases and International Health, University of Virginia School of Medicine, P.O. Box 801340, Charlottesville, VA 22908 USA; 2 Division of Disease Prevention, TB Control, Virginia Department of Health, 109 Governor Street, Richmond, VA 23219 USA; 3 TB Control and Newcomer Health, Virginia Department of Health, PO Box 2448, 109 Governor Street, Room 326, Richmond, VA 23218 USA

**Keywords:** Tuberculosis, Diabetes, Sputum culture conversion, Therapeutic drug monitoring, Clinical outcomes, Early intervention

## Abstract

**Background:**

Diabetes complicates tuberculosis (TB) treatment including a prolonged time of sputum culture conversion to negative growth. Since 2013 in Virginia, interventions early in the treatment course have used therapeutic drug monitoring and dose correction for isoniazid and rifampin after 2 weeks of TB treatment in patients with diabetes along with nurse manager initiated diabetes education and linkage to care.

**Methods:**

A retrospective cohort study of the state TB registry was performed for patients initiating drug-susceptible pulmonary TB treatment that were matched for age, gender, chest imaging and sputum smear status to compare time to sputum culture conversion and other clinical outcomes in the pre-and post-intervention groups.

**Results:**

Three hundred sixty-three patients had documented time to sputum culture conversion in the pre-and post-intervention periods, including 56 (15%) with diabetes. Seventy-four (57%) of all patients with diabetes were ≥60 years of age at treatment initiation. Twenty-six patients with diabetes were matched in each group. Mean time to sputum culture conversion in the post-intervention group was 42 ± 22 days compared to the pre-intervention group of 62 ± 31 days (*p* = 0.01). In the post-intervention group 21 (80%) of patients with diabetes had culture conversion by 2 months compared to 13 (50%) in the pre-intervention group (*p* = 0.04).

**Conclusions:**

Early interventions for diabetes related TB in the programmatic setting may hasten sputum culture conversion.

## Background

Tuberculosis (TB) was responsible for 1.5 million deaths worldwide in 2014 [1]. Global estimates suggest 15% of all patients with TB also have diabetes mellitus and the overall prevalence of diabetes is increasing in TB-endemic regions [2]. Diabetes not only increases the risk of developing active TB by 3-fold [3–6], but also patients with diabetes are more likely to have poor microbiological and clinical outcomes when developing active TB disease compared to those without diabetes, such as a delayed time to sputum culture conversion to negative and an increased risk of death [7, 8]. Despite these associations most initiatives have focused on screening strategies for diabetes among TB patients, but few have targeted improving treatment outcomes once diabetes related TB has been diagnosed.

In 2014, in the state of Virginia, USA, one out of 11 people was diagnosed with diabetes at a mean age of 47.6 ± 1.0 years [9]. Additionally, in 2014 the state had a TB case rate of 2.4 per 100,000 persons, which was a 10% increase from the prior year. Of those TB cases, 16.7% were reported to have diabetes, a proportion which had steadily increased over the last decade [10]. In an earlier study from Virginia, TB patients with diabetes were 7 times more likely to have slow response to therapy compared to those without diabetes. Slow response was defined as persistence of TB-specific symptoms, no decrement in acid-fast quantification by smear microscopy or radiographic changes that were worsening after 1 month or more of treatment [11]. That study also noted among slow responders that those with diabetes had lower concentrations of rifampin compared to those without diabetes. Lower drug concentration in diabetes patients may be related to impaired drug absorption secondary to either delayed gastric emptying or hyperglycemia which may influence gastric hydrochloric acid secretion [12, 13]. While therapeutic drug monitoring (TDM) had been more routinely used in patients after the development of slow response, these findings led to increasing use of TDM earlier in the course of therapy in an effort to prevent slow response in diabetes related TB. Hence, in 2013 statewide recommendations were updated to perform TDM at 2 weeks after treatment initiation for all patients with diabetes, so-called early TDM, a process found to be feasible for the majority of all cases in the state, and during which 21 (76%) of all diabetes patients tested had peak serum concentrations of isoniazid, rifampin or both that were below the expected range [14, 15]. At the same time, other interventions aimed at this subpopulation included more active laboratory screening for diabetes among TB patients initiating therapy, and development of an educational flipchart adapted for nurse to patient counseling during directly observed therapy to reinforce healthy living strategies for diabetes and assure linkage to diabetes care [16, 17].

Therefore, we sought to compare clinically relevant outcomes among patients with diabetes initiating TB treatment in the state before and after the implementation of these early interventions. The pre-and post-intervention change in outcomes were also compared between patients with and without diabetes. Given that mortality rates from TB are low in the state, the primary outcome of interest was sputum culture conversion to negative. Lack of conversion by 2 months has been used as a predictor of relapse, for example in one study correlating with a relapse rate of 10% within 1 year [18], and the time to culture conversion may influence a clinician’s determination of total treatment duration [19–21].

## Methods

### Subjects

A retrospective cohort analysis was performed among patients that were newly diagnosed with active TB and started on anti-TB therapy during the period of January 2009- December 2010 (pre-intervention) and January 2013–December 2014 (post-intervention) in the state. These dates were used as early TDM for patients with diabetes was occasionally employed during the years of 2011–2012 prior to formal recommendations. Data were collected for all patients ≥18 years old from the state TB registry which captures all treatment initiation. The study was approved by the institutional review board at the University of Virginia and the Virginia Department of Health.

Surveillance data for the pre-intervention group (2009–2010) and post-intervention group (2013–2014) included demographics, co-morbidities including human immunodeficiency virus (HIV) infection and diabetes, prior TB history and anatomic focus of the current TB episode categorized as pulmonary, extra-pulmonary or both. Laboratory report forms for patients with TDM were reviewed when available. Diabetes diagnosis was determined by self-report for patients already on anti-diabetes treatment, or by report of caregivers to nurse managers or review of medical charts. Laboratory results for diabetes diagnosis or disease severity, such as glycosylated hemoglobin (HbAlc) or fasting blood glucose, were not required for reporting in the registry.

### Procedures

In Virginia, all cases of active TB are reported to the Virginia Department of Health and assigned to a nurse manager. Directly observed therapy is administered by the nurse manager or a trained outreach worker. In the pre-intervention time period, TDM was recommended to be performed only among those patients that demonstrated slow response usually 8 weeks or more after treatment initiation. The standard procedure for TDM was to directly administer medication and then collect venous blood 2 h later at the time of estimated C_max_. After collection, serum was separated by centrifugation at the local health department and transported on dry ice to the referral laboratory at the University of Florida where validated high performance liquid chromatography or gas chromatography results were available within 48 h and reported in reference to the expected μg/mL range [22]. The interpretation of TDM and the management of slow-responders were referred to state TB consultants.

In the post-intervention time period, slow-responders were managed similarly, but additionally all patients identified as having diabetes were recommended for TDM. TDM was performed at approximately 2 weeks after treatment initiation to allow steady state metabolism of anti-TB drugs. In contrast to those with slow response where dose adjustment after TDM was often individualized and directed by the state TB consultants, for those patients with diabetes and early TDM in whom concentrations were below the expected C_max_ range (for isoniazid 3–5 μg/ml or rifampin 8–24 μg/ml), doses were adjusted by a single dose increase. For example, recommendations stated if the patient was prescribed a daily dosed rifampin of 600 mg and the drug concentration was below the expected range, the dose was increased to 900 mg; and for daily dosed isoniazid of 300 mg, the dose was increased to 450 mg [11, 15]. Complications with dose adjustment or major toxicity were reported to the state TB control program. Following early TDM, patients were further monitored for slow response, and if later identified as slow responders then referral made to a state TB consultant. In the post-intervention period recommendations were made to nurse managers to interview all patients with diabetes utilizing the educational flipchart and assess the patient’s understanding of diabetes related TB and linkage to diabetes care. Documentation of the use of the flipchart or linkage to care was not available in the state TB registry.

Per routine in both pre and post-intervention periods, patients with pulmonary TB had sputum collected weekly until smear microscopy conversion to negative and then at least monthly thereafter until culture conversion. Time to culture conversion was recorded as the date of collection of the first of two consecutive sputum samples without mycobacterial growth and calculated in days from the start of TB treatment initiation.

### Statistical analysis

Demographic and clinical characteristics were compared between all patients in pre-and post-intervention groups by the *χ*2 statistic or for continuous variables, the Student t-test or the Mann–Whitney U test when appropriate. The time to sputum culture conversion and the proportion with culture conversion before 8 weeks were analyzed among the subgroups of patients with pulmonary TB for whom a time to culture conversion was documented. Patients were excluded from these analyses if their *M. tuberculosis* isolate was resistant to isoniazid, rifampin, pyrazinamide or ethambutol or if they were treated with any other second-line drugs. The mean time to sputum culture conversion and the proportion with culture conversion before 8 weeks were compared by the student t-test and *χ*2 statistic respectively.

**Fig. 1:**
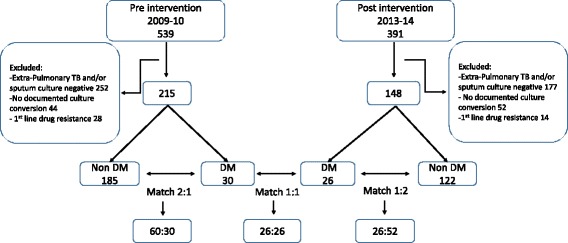
Flowchart of patients included for analysis; DM = Diabetes mellitus

To account for the non-randomization of the retrospective cohort and to minimize potential selection bias, non-diabetes and diabetes patients were matched 2:1 on basic demographics or clinical characteristics previously reported to influence culture conversion including age (±15 years), gender, chest imaging with or without cavitary lesions and sputum smear status (positive or negative at treatment initiation). Non-diabetes and diabetes patients had time to culture conversion and 8-week culture conversion compared separately in both the pre and post-intervention groups. Lastly, to restrict analysis to known diabetes patients alone, these outcomes were compared between the pre and post-intervention groups but due to the smaller numbers of diabetes patients, matching could only be performed in a 1:1 allocation (Fig. 1). The matching process and all other statistical analyses were completed using IBM SPSS statistics for Windows, version 23.0 (IBM Corp, Armonk, NY).

## Results

In total, 539 patients in the pre-intervention period and 391 patients in the post-intervention period were initiated on TB treatment, including 64 (12%) with diabetes in the pre-intervention period and 66 (17%) in the post-intervention period. Including patients from both groups, those with diabetes had a mean age of 61 ± 15 years and were older than those without diabetes 41 ± 20 years (*p* < 0.001). Indeed, 74 (57%) of all patients with diabetes were ≥60 years of age at treatment initiation. There were no other differences in baseline patient demographics between the pre and post-intervention periods (Table 1).

**Table 1:** Baseline characteristics for patients treated for tuberculosis

Characteristic No. (%)	All patients *N* = 930			DM *N* = 130		
	2009-10 *N* = 539	2013-14 *N* = 391	*p*-value	2009-10 *N* = 64	2013-14 *N* = 66	*p*-value
Age (years, mean ± SD)	43 ± 21	45 ± 21	0.11	60 ± 18	63 ± 13	0.32
Male	309 (57)	216 (55)	0.55	35 (55)	43 (65)	0.28
HIV positive	19 (4)	20 (5)	0.32	0	1 (2)	1.00
Diseases site						
Pulmonary only	359 (67)	269 (69)	0.19	53 (83)	54 (82)	0.99
Extra-Pulmonary only	100 (19)	87 (22)	0.67	11 (17)	12 (18)	0.87
Both	80 (15)	35 (9)	0.03	5 (17)	7 (11)	0.55
Sputum culture positive	287 (53)	214 (55)	0.43	36 (56)	40 (61)	0.44
Sputum smear positive	215 (40)	161 (41)	0.15	33 (52)	32 (49)	0.68
Cavitary lesion	181 (34)	131 (34)	0.89	27 (42)	29 (44)	0.86
INH Resistant	43 (10)	26 (9)	0.99	2 (4)	4 (8)	0.42
RIF Resistant	3 (1)	6 (2)	0.70	0	1 (2)	0.47

### Outcomes among patients with sputum culture conversion

Including patients from the pre and post-intervention periods, 363 met criteria (confirmed drug-susceptible pulmonary TB with documented sputum culture conversion) for outcomes analyses including 56 (15%) with diabetes (Fig. 1). Among all patients, the time to culture conversion was significantly improved from the pre-intervention group, 56 ± 35 days, to the post-intervention group, 43 ± 28 days (*p* < 0.001) (Table 2). While this change remained significant when restricted to non-diabetes patients that converted 8 days earlier in the post compared to the pre-intervention period, the improvement in time to culture conversion was greater for the diabetes patients with the post-intervention group that converted on average 19 days earlier (*p* = 0.02). The same trends were found for culture conversion before 8 weeks where for example the proportion among those with diabetes increased from only 15 (50%) in the pre-intervention group to 21 (80%) in the post-intervention group (*p* = 0.03). The death rate was not significantly different between the groups (Table 2).

**Table 2:** Clinical outcomes of adults with drug-susceptible tuberculosis and documented sputum time to culture conversion in days

Outcome	All patients *N* = 363			Non DM *N* = 307			DM *N* = 56		
	2009-10 *N* = 215	2013-14 *N* = 148	*p*-value	2009-10 *N* = 185	2013-14 *N* = 122	*p*-value	2009-10 *N* = 30	2013-14 *N* = 26	*p*-value
Time to culture conversion (days, mean ± SD)	56 ± 35	43 ± 28	<0.001	51 ± 36	43 ± 30	0.003	61 ± 32	42 ± 22	0.02
2 months culture conversion No. (%)	126 (59)	110 (74)	0.002	111 (60)	89 (73)	0.02	15 (50)	21 (80)	0.03
Death No. (%)	5 (2)	2 (1)	0.71	4 (2)	1 (1)	0.65	1 (3)	1 (4)	1.00

### Outcomes among matched cases

**Fig. 2:**
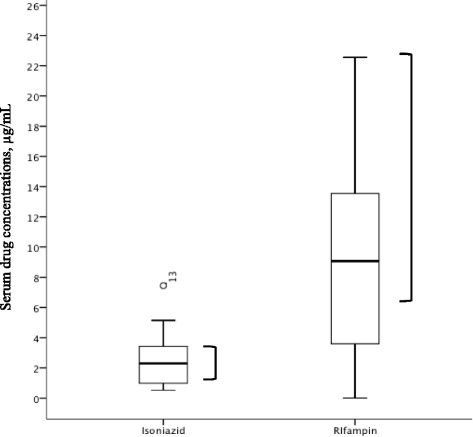
C_2hr_ results for early therapeutic drug monitoring among diabetes patients in the post-intervention group. Boxes= interquartile ranges with median line, whiskers= minimum and maximum values, and one circle (for isoniazid) is a statistical outlier. Brackets represent the expected range for the Cmax; isoniazid 3-5 μg/ml and rifampin 8-24 μg/ml

When matching non-diabetes and diabetes cases (2:1) and comparing separately these subgroups within the pre-intervention and post-intervention periods, patients with diabetes in the post-intervention group converted earlier than the non-diabetic patients in the same group (*p* = 0.08) and even earlier than both diabetes and non-diabetes patients in the pre-intervention group (Table 3). Matched comparison of patients with diabetes only in the pre and post-intervention groups (1:1) found on average a 20 days’ earlier time to sputum culture conversion (62 ± 31 vs 42 ± 22 days; *p* = 0.01) (Table 4). Only 17 of the 26-matched diabetes had TDM results available for review, but of those 12 (71%) had a concentration of rifampin or isoniazid in the range of dose adjustment (Fig. 2). Those 17 subjects had time to culture conversion 20 days earlier when compared to matched diabetes patients in the pre-intervention without TDM (40 ± 17 vs 63 ± 33; *p* = 0.02). Of note, no major adverse effects from dose adjustment were reported to the state TB control program, but final regimens and doses were not available for review.

**Table 3:** Sputum culture conversion in adults with pulmonary tuberculosis matched 2:1 non-diabetes to diabetes for age, gender, sputum smear result and chest x-ray findings

Outcome	Matched non DM:DM 2009-2010			Matched non DM:DM 2014-15		
	non DM *N* = 60	DM *N* = 30	*p*-value	non DM *N* = 52	DM *N* = 26	*p*-value
Time to culture conversion (days, mean ± SD)	57 ± 35	61 ± 32	0.62	57 ± 37	42 ± 22	0.08
2 months culture conversion, No. (%)	34 (57)	15 (50)	0.55	31 (60)	21 (81)	0.12

**Table 4:** Sputum culture conversion in only diabetic patients only matched 1:1 pre and post intervention for age, gender, sputum smear result and chest x-ray findings

Outcome	2009-10 *N* = 26	2013-14 *N* = 26	*p*-value
Time to culture conversion (days)	62 ± 31	42 ± 22	0.01
2 months’ culture conversion No. %	13 (50)	21 (80)	0.04

## Discussion

This retrospective cohort study assessed the impact of early interventions among patients with diabetes related TB and found improvement in microbiological clearance from the sputum in all patients between the pre and post-intervention periods but the improvement was more pronounced in those with diabetes. These findings contrast to most non-interventional studies that note a delay in time to sputum culture conversion to negative or other worse outcomes in patients with diabetes compared to those without diabetes [6, 17]. While we detected no difference in time to sputum culture conversion in the pre-intervention period between matched patients with and without diabetes, we found a decrease in time to sputum culture conversion among patients with diabetes compared to those without diabetes in the post-intervention period.

The validity of these observations was strengthened by the complementary matched analyses. Importantly, when patients with known diabetes were matched from the pre and post-intervention periods, time to sputum culture conversion decreased significantly by an average of 20 days in the post-intervention group and the proportion converting by 8 weeks increased to 80%. Given the relationship of delayed culture conversion to relapse of disease or the practice of extending treatment duration for those that fail to culture convert by 8 weeks and have cavitary lung disease [21, 23], these findings may be clinically meaningful.

The improvement in microbiological outcomes for diabetes patients in the post-intervention period are suggestive that the interventions early in the treatment course led to this change. However, quantifying the effects of these individual interventions remains challenging. Enhanced case finding by more routine use of laboratory diagnostics such as HbA1c testing, would have identified diabetes that could have been undiagnosed in the pre-intervention period. Additionally, this may explain the proportional increase in diabetes patients among all TB cases between the pre and post-intervention periods. Yet, during the study periods, HbA1c testing was not required for reporting in the state registry, although now an initiative is in place to make this testing available for all TB patients in the state. Diagnosis alone would not improve treatment outcomes unless patients with diabetes were treated differently, and there is emerging evidence that metformin may have anti-TB effects independent of control of hyperglycemia by means of enhancing autophagy, a form of mycobacterial killing by the host [24]. Unfortunately, the surveillance data did not allow for detail of the diabetic regimens.

It is also plausible that early TDM accounted for the hastening in sputum culture conversion. A recent prospective study from a TB endemic region demonstrated that individual pharmacokinetic variability was the primary driver of treatment failure [25]. Even in the randomized controlled trial setting, higher doses of rifapentine substituted for rifampin in the first-line therapy for drug susceptible TB, time to culture conversion was related to rifapentine exposure as measured by the area under the time-concentration curve (AUC) from a full pharmacokinetic sampling interval [26]. Future study of rifapentine will use an AUC informed fixed dose not because an individual’s drug exposure was unimportant but because TDM is believed to be impractical for TB endemic settings. Barriers to TDM implementation are highlighted in a recent meta-analysis that aimed to summarize the evidence for the use of TDM but found generally low-quality observational studies from a few specialized centers [27]. Still other individual studies from well-resourced settings which have concluded that TDM did not affect outcome have examined its use only after patients have manifested slow response to therapy where equivalence in outcome could instead be interpreted as an actual benefit conferred by TDM in preventing the more rare events of relapse or acquired drug resistance [28]. In contrast in Virginia, recommendations were made to use TDM systematically early in the treatment course prior to the development of slow response for a distinct subpopulation at higher risk of poor treatment outcome.

Earlier studies of TDM are also limited in their heterogeneity of what drugs were assayed, what assays were used for measurement and what concentrations were used to prompt dose increase [29]. Even though isoniazid and rifampin both kill *M. tuberculosis* in a concentration dependent manner, single or limited time points as estimates of C_max_ may imprecisely approximate the AUC and the lower limits of the expected ranges for these time points are extrapolated from smaller controlled studies of TB patients or from healthy controls. Certainly further study is needed to define pharmacokinetic thresholds associated with outcome among a diversity of patient populations with active TB [30] and relative to minimum inhibitory concentrations. Our findings would suggest that despite these inherent imprecisions, TDM when performed with consistent procedures [11, 12] may provide a clinically actionable result within a programmatic setting.

Older age diabetes patients comprise a growing proportion of TB patients in Virginia, an epidemiological trend we expect will be increasingly mirrored in other locations as the global burden of diabetes expands and dietary and/or lifestyle practices westernize among TB prevalent communities. Without understatement, this trend represents an emerging threat to TB control which has prompted global stakeholders to call for a response that capitalizes on the lessons learned from HIV related TB [31]. For example, in Taiwan, the Bureau of National Health Insurance has implemented a pay-for-performance program for patients with diabetes and found a significant decrease in the development of active TB compared to diabetes patients not enrolled in the program and a decrease in death from TB during treatment relative to diabetes patients not enrolled and non-diabetes patients [32]. Strategies that have integrated HIV and TB diagnostics and therapy have achieved remarkable success in the era of antiretroviral rollout even for drug-resistant TB [33, 34], and similarly bold approaches may be necessary for diabetes related TB.

There are several other limitations in this study, given the retrospective, non-randomized design and inability to capture definitive laboratory data on all patients including those that were categorized as not having diabetes when laboratory diagnostics for diabetes may simply not have been performed. However, this potential misclassification would not have affected the matched comparison of patients with known diabetes pre and post-intervention. While the improvement in time to culture conversion was maintained when the analysis was restricted to those with known early TDM performed, clinicians may not have followed the recommendations for dose adjustment and the influence of the final dose or regimen could not be estimated. It is also possible that those patients with diabetes that had early interventions were monitored more intensively with more frequent sputum samples but this would be unlikely in a programmatic setting and the magnitude of improvement in the diabetes patients from pre to post-intervention would not be expected from more frequent sampling alone. As markers of diabetes disease severity were not routinely measured in the registry, it remains possible that post-intervention patients with diabetes had less severe stages of diabetes disease that could have accounted for some of the improved clinical response. Other confounders were not immediately apparent.

## Conclusions

In summary, early interventions for diabetes related TB in the programmatic setting hasten sputum culture conversion but require validation in other diabetes prevalent populations, including further prospective study on the relative impact of early TDM and its effect on other longer term clinical outcomes such as treatment duration, relapse or with regard to TB endemic settings, early treatment failure. At a minimum, these findings highlight the need for dedicated attention to this emerging co-epidemic.

## Abbreviations

AUC: Area under the concentration curve
DM: Diabetes mellitus
HIV: Human immunodeficiency virus
TB: Tuberculosis
TDM: Therapeutic drug monitoring
